# Sacubitril/Valsartan Improves Cardiac Function in Dialysis Patients

**DOI:** 10.7759/cureus.63360

**Published:** 2024-06-28

**Authors:** Zaher Armaly, Amer Saffouri, Habib Kordahji, Munir Hamzeh, Elias Bishouty, Narmin Matar, Maha Zaher, Adel Jabbour, Dahud Qarawani

**Affiliations:** 1 Nephrology, Edinburgh Medical Missionary Society (EMMS) Nazareth Hospital, Nazareth, ISR; 2 Internal Medicine, Edinburgh Medical Missionary Society (EMMS) Nazareth Hospital, Nazareth, ISR; 3 Cardiology, Clalit Health Services, Nazareth, ISR; 4 Surgery, Carmel Medical Center, Haifa, ISR; 5 Biomedical Laboratory, Edinburgh Medical Missionary Society (EMMS) Nazareth Hospital, Nazareth, ISR; 6 Cardiology, Edinburgh Medical Missionary Society (EMMS) Nazareth Hospital, Nazareth, ISR

**Keywords:** end-stage renal disease (esrd), heath-related quality of life, heart failure with reduced ejection fraction, dialysis, sacubitril/valsartan

## Abstract

Heart failure (HF) is characterized by the activation of adverse neurohormonal systems and a high mortality rate. Noteworthy, HF is a well-known complication of chronic kidney disease (CKD), especially in end-stage kidney disease (ESKD), where dialysis patients are seven to eight times more likely to encounter cardiac arrest than the general population. Therefore, it is important to develop efficient treatments to improve cardiac function in dialysis patients and eventually reduce the cardiovascular death toll. Sacubitril/valsartan (Sac/Val) is a dual inhibitor/blocker of the neprilysin and angiotensin II receptors, which exert cardioprotective effects among patients with heart failure with reduced ejection fraction (HFrEF) and heart failure with preserved EF (HFpEF). Unfortunately, the drug is not approved for subjects with advanced CKD or dialysis patients due to safety concerns. The current study examined the cardiac effects of Sac/Val in HD patients. Administration of Sac/Val (100-400 mg/day) to 12 hemodialysis (HD) patients with HFrEF for six months gradually improved ejection fraction (EF) independently of morphological changes in cardiac geometry, as assessed by echocardiography (ECHO), and hemodynamic alterations. Interestingly, the Cardiomyopathy Questionnaire (Kansas City KCCQ-12) revealed that quality of life significantly improved after Sac/Val treatment. No major adverse effects were reported in the present study, supporting the safety of Sac/Val at least in these patients and for the applied follow-up period. Collectively, these findings support the use of Sac/Val as a cardioprotective agent in both HD and peritoneal dialysis (PD) patients.

Yet, a more comprehensive study is required to establish these findings and to extend the follow-up period for 12 months in order to solidify these encouraging results.

## Introduction

Sacubitril/valsartan (Sac/Val) is a dual inhibitor/blocker of the neprilysin and angiotensin II receptors, respectively [1]. Sacubitril/valsartan has been shown to exert beneficial cardiovascular effects, including improving the prognosis of heart failure (HF) patients and reducing pulmonary hypertension (PAH) [2-4]. Concerning HF, since its introduction two decades ago, Sac/Val has become a key drug for the treatment of heart failure with reduced ejection fraction (HFrEF) [2, 3, 5, 6]. Specifically, several reports have demonstrated that Sac/Val significantly reduced cardiovascular mortality and hospitalization due to worsening HF among HFrEF patients, as compared to an angiotensin-converting enzyme inhibitor (ACEi) [7]. Besides counteracting the adverse cardiorenal effects of the upregulated renin-angiotensin-aldosterone system (RAAS) via angiotensin 1 receptor (AT1R) blockade, inhibition of neprilysin by sacubitril increases natriuretic peptides (NPs) by preventing their degradation, thus inducing natriuresis, reduction of blood pressure, inhibition of cardiac myocyte hypertrophy, apoptosis, and fibrosis [6]. At the pulmonary level, Burgdorf et al. reported that Sac/Val reduced pulmonary artery pressure (PAP) and mean pulmonary capillary wedge pressure (PCWP) from 51 mmHg and 21 mmHg to 44 and 16 mmHg, respectively, in patients with heart failure with preserved EF (HFpEF) [4]. Moreover, clinically, the New York Heart Association functional class improved in 12 of the 18 patients when placed on Sac/Val. Interestingly, echocardiographic analysis of left ventricular function was not changed post-Sac/Val treatment. In addition, improved PAH in patients with HFpEF was observed following Sac/Val treatment [4]. Similarly, it has been shown that Sac/Val reverses pulmonary vascular remodeling and reduces pulmonary artery systolic pressure in patients with HFpEF [8]. However, whether Sac/Val is cardioprotective in dialysis patients with HFrEF (ejection fraction (EF)<40%) is largely unknown. Therefore, the current study examines the efficacy of Sac/Val in dialysis patients with advanced HF. This question was not addressed previously, as Sac/Val is not approved for hemodialysis (HD) patients due to safety concerns [9]. We were encouraged by the study of Guo et al. [10], who applied Sac/Val in 71 HD patients, where this drug converted pulmonary artery systolic pressure (PASP) after three months of administration. In addition, this therapeutic protocol improved right atrial diameter (RAD), left ventricular diameter (LVD), left ventricular posterior wall thickness (LVPWT), left atrial diameter (LAD), pulmonary artery diameter (PAD), left ventricular end-diastolic volume (LVEDV), left ventricular end-systolic volume (LVESV), left ventricular ejection fraction (LVEF), and fractional shortening (FS) as compared to angiotensin receptor blockers (ARBs). In line with its cardioprotective effects, b-type natriuretic peptide (NT-proBNP) and cardiac troponin I (cTnI) levels were significantly reduced following Sac/Val treatment. Noteworthy, the drug was safe in these patients, and adverse events were not observed.

This article was previously presented as an abstract at the 2023 American Society of Nephrology (ASN) Annual Scientific Meeting in Philadelphia, PA, on November 2-5, 2023.

## Materials and methods

We conducted this prospective study on both HD and peritoneal dialysis (PD) patients who were prescribed HD and PD treatment in the Department of Nephrology at Nazareth Hospital Edinburgh Medical Missionary Society (EMMS) Nazareth, Israel. The study was approved by the Nazareth Hospital EMMS Human Research Review Committee (approval number: 22-21-EMMS) and carried out at Nazareth Hospital. All patients provided informed consent. Inclusion criteria include chronic dialysis (more than six months), either HD or PD and HFrEF (EF<40%). The number of HD and PD patients with HFrHF in the Nazareth EMMS unit that were recruited was 12 of 140 and one of 15 patients, respectively. All HD and PD patients received conventional dialysis regimens (three sessions a week for HD; daily dialysis for PD), and all received conventional treatment for HF (including RAAS inhibitors) and other background illnesses. Eligible patients (n = 13 in total) were treated with Sac/Val at incremental doses of 2x50 mg/day up to 2x200 mg/day.

Demographic data and blood samples were collected, and an echocardiogram (ECHO) was performed after three and six months. Hematological parameters included CBC, the full chemistry panel, glycated hemoglobin (HBA1C), parathormone (PTH), and NT proBNP, which were measured before and after treatment. The ECHO was performed by two cardiologists who double-checked each other. Echocardiographic indices included LVPWT, LAD, LVEDV, LVESV, LVEF, and PASP. Creatinine clearance (CCT) in patients with residual renal function (RRF) was assisted before the study's initiation and three and six months later.

Quality of life

The Cardiomyopathy Questionnaire (Kansas City KCCQ-12) was applied to assess changes in quality of life after Sac/Val as compared to pretreatment status (Appendix A).

Major adverse events

Major adverse cardiac events (MACE), hospital admissions due to HF, the need for extra dialysis, changes in peritoneal fluid concentration as well as the number of daily exchanges, changes in the intradialytic body weight, hemodynamic changes (Bp), improvement in quality of dialysis (KT/V), and CCT were assisted.

Statistical analysis

Data are expressed as means ± standard error of mean (SEM). Microsoft Excel software (Microsoft Corp., Redmond, WA) was used to analyze data, create standard curves, and draw figures. The statistical significance between post-treatment and pre-treatment was assessed by a t-test. P-value ≤0.05 was considered statistically significant.

## Results

Baseline characteristics

The demographic and laboratory data of the studied patients, including age and sex, such as sex, age, dialysis access, duration of dialysis, background diseases, and applied drugs, are listed in Table 1. The average age of the studied patients was 66.5 ± 3.0 years (Table 1). The male/female ratio was 85/15%. The average systolic blood pressure (SBP) and diastolic blood pressure (DBP) were 143 ± 7 and 63 ± 3 mmHg, respectively (Table 1).

**Table 1:** Demographic and clinical characteristics of the patients in the study group M: male; F: female; AV: aortocaval; DM: diabetes mellitus; IHD: ischemic heart disease; CHF: congestive heart failure; HTN: hypertension; PVD: peripheral vascular disease; HPL: hyperlipidemia; APKD: PVD: TIA: transient ischemic attack; CVA: cerebrovascular accident; AF: atrial fibrillation; CVC: central venous catheter

Patient #	Gender	Age (y)	SBP (mmHg)	DBP (mmHg)	Dialysis access	Dialysis duration (y)	Background diseases
1	M	48	65	125	Native AV-fistula	6	DM, IHD, CHF, HTN, PVD, HPL
2	F	66	77	120	Native AV-fistula	7	APKD, HTN, CHF
3	F	62	52	105	Gortex	6	DM, HPL, HTN, CHF
4	M	63	50	112	Gortex	15	DM, HPL, CHF, IHD
5	M	79	53	134	Native AV-fistula	3	HTN, CHF, hypercholesterolemia, diverticulosis
6	M	84	43	154	Native AV-fistula	8	DM, HTN, HPL
7	M	74	67	169	CVC	1.3	DM, IHD, CHF
8	M	76	77	156	CVC	5	HTN, IHD, PVD
9	M	56	53	121	Native AV-fistula	4	Polycystic kidney, AF, HTN
10	M	72	72	157	Native AV-fistula	12	DM, IHD, HTN, HPL, PVD
11	M	51	78	170	Native AV-fistula	3	DM, HTN, IHD, TIA
12	M	61	73	145	CVC	0.66	HTN, DM, HYP, CHF, CVA
13	M	68	65	133	Tenckhoff	0.45	DM, IHD, CHF, HTN

**Figure 1:**
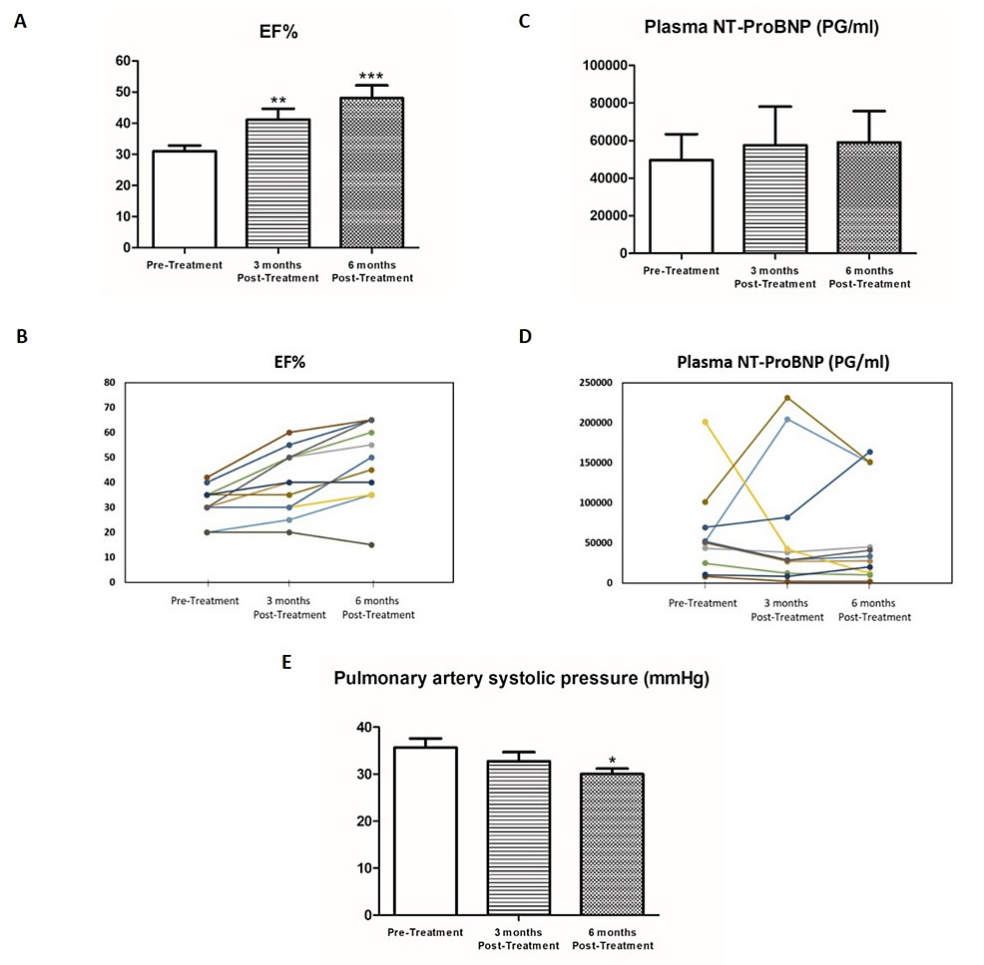
Effects of sacubitril/valsartan on cardiac, biochemical, and hemodynamic parameters in dialysis patients with EF<40% pre- and post-treatment for three and six months. Effects of sacubitril/valsartan on ejection fraction (EF) (A&B), b-type natriuretic peptide (NT-proBNP) (C&D), and pulmonary artery systolic pressure (PASP) (E) in hemodialysis (HD) and peritoneal (PD) patients with EF <40% pre- and post-treatment for three and six months. *P <0.05; **P <0.01; ***P <0.001

The EF was 32.1 ± 1.15% at baseline and increased to 41.15 ± 1.78% (P <0.01) and 48.63 ± 4.72 (P <0.005) following three and six months of treatment with Sac/Val, respectively (Figure 1A). Improvement in EF was obtained in 10 out of 12 HD patients, but not in PD patients who were treated with the drug (Figure 1B). The non-responder patients were characterized by global hypokinesis and low EF (20%-30%). Besides EF, we measured NT-proBNP, and no significant changes in this biomarker were observed following Sac/Val (Figures 1C, 1D). Basal NT-proBNP levels were 59,624 ± 13,812 pg/ml before treatment and remained stable after three and six months after the drug administration (57,566 ± 20,592 and 59,124 ± 16,568 pg/ml, respectively, P = not significant (NS)).

Echocardiography was performed prior to dialysis and three and six months after Sac/Val treatment by two experienced cardiologists from our two different cardiologic units independently and blindly to the patients included in the study. The comparison of laboratory and ECHO data pre- and post-treatment are summarized in Table 2. There were no differences in basal biochemical and hematological parameters such as hemoglobin, hematocrit, serum potassium, serum calcium, serum phosphorus, serum albumin, and parathyroid hormone and those measured post-Sac/Val treatment (Table 2). Administration of Sac/Val for three and six months significantly lowered PASP. However, no improvements in the RAD, LVD, LVPWT, LAD, PAD, LVEDV, LVESV, or LVEF were detected following Sac/Val treatment. Interestingly, HBA1C levels unexpectedly improved in 80% of patients receiving the drug without change in the antidiabetic drugs (Table 2).

**Table 2:** Echocardiographic parameters pre- and post-six months of treatment Hb: hemoglobin; Hct: hematocrit; K+: potassium; Ca++: calcium; PO4: phosphorous; Alb: albumin; PTH: parathyroid hormone LVPWT: left ventricular posterior wall thickness; LAD: left atrial diameter; LVEDV: left ventricular end-diastolic volume; LVESV: left ventricular end-systolic volume; LVEF: left ventricular ejection fraction; PASP: pulmonary artery systolic pressure.

Parameter	Pre-treatment	Post-treatment	P-value
Hb (gr%)	11.34±0.26	10.95±0.3033	0.3384248
Hct (%)	34.83±0.89	33.98±1.03	0.5228363
K+ (mmol/l)	5.16±0.14	5.24±0.18	0.7431692
Ca++ (mg/dl)	8.43±0.13	8.17±0.19	0.2600647
PO4 (mg/dl)	5.11±0.33	5.17±0.35	0.9139451
Alb (gr%)	3.81±0.10	3.83±0.12	0.9246749
PTH (pg/ml)	640.15±128.6	639.08±140	0.9955275
LVPWT (mm)	1.06±0.22	1.082±0.026	0.64
LAD (mm)	4.4±0.22	4.09±0.2	0.327
LVEDV (ml)	156.4±16.05	131.8±16.5	0.31
LVESV (ml)	101.45±10.7	81.82±14.9	0.316
LVEF (%)	32.9±1.78	48.63±4.72	0.00547
PASP (mmHg)	35.63±0.59	30±0.35	0.022

Quality of life

**Figure 2:**
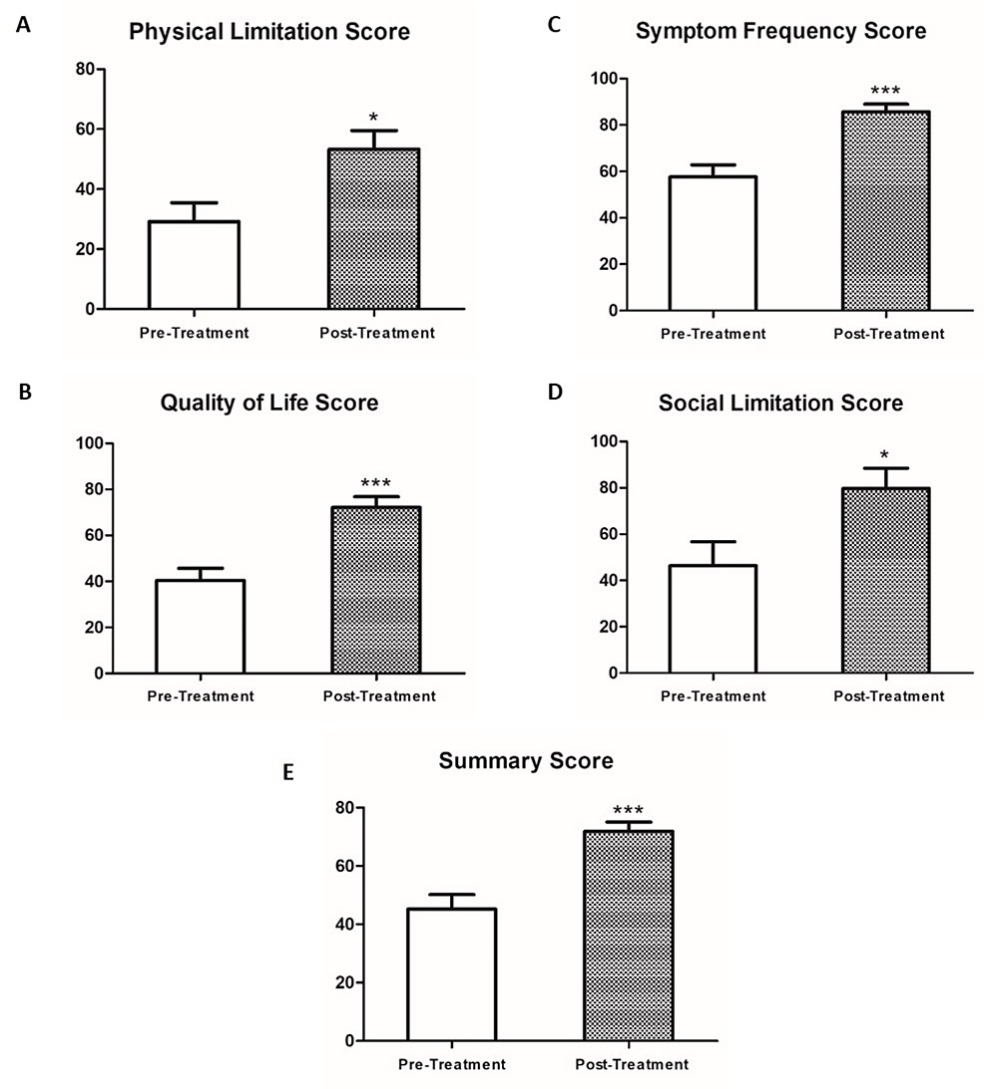
Effects of sacubitril/valsartan on physical limitations, symptoms, quality of life, and social limitations in dialysis patients for six months Effects of sacubitril/valsartan on physical limitations, symptoms, quality of life, and social limitations in hemodialysis (HD) and peritoneal dialysis (PD) patients with an ejection fraction (EF) <40% pre- and post-treatment for three and six months. *P <0.05; **P <0.01; ***P <0.001

Applying the Cardiomyopathy Questionnaire (Kansas City KCCQ-12) revealed that most of the patients who were treated with Sac/Val reported substantial improvement in their well-being and quality of life (Table 3, Figure 2). Specifically, walking distance and dyspnea improved in two-thirds of the treated patients, whereas foot swelling decreased in half of the patients. An overwhelming majority of patients reported less fatigue and enhanced social activity and life enjoyment as compared to the pre-treatment period. 

**Table 3:** Effects of sacubitril/valsartan on physical limitations, symptoms, quality of life, and social limitations in dialysis patients Effects of sacubitril/valsartan on physical limitations, symptoms, quality of life, and social limitations in hemodialysis (HD) and peritoneal dialysis (PD) patients with an ejection fraction (EF) <40% pre- and post-treatment for six months

Item	Pre-treatment	Post-treatment	P-value (pre-treatment vs. post-treatment)
Physical limitation score	29.13±6.24	53.29±6.23	0.013
Symptom frequency score	57.6±5.19	85.73±3.19	0.00011
Quality of life score	40.38±5.32	72.11±4.72	0.00016
Social limitation score	46.41±10.26	79.74±8.7	0.029
Summary score	45.15±5.04	71.84±3.21	0.00016

Adverse events

One patient experienced dizziness after the second dose, with no changes in hemodynamic parameters. No MACE or hospital admissions were observed during the three-month follow-up period.

## Discussion

To the best of our knowledge, this is the second study to evaluate the efficacy of Sac/Val in dialysis patients with HF. Our findings clearly show that Sac/Val improves cardiac output and is safe in both HD and PD patients, supporting the use of this drug in end-stage kidney disease (ESKD) accompanied by HF without fear of adverse events.

Despite the well-established cardioprotective effects of Sac/Val in patients with HFrEF, the use of this drug in patients with advanced chronic kidney disease (CKD) and ESKD on dialysis was not approved due to potential deleterious renal effects and safety concerns in this subgroup of patients [9], as well as the risk of ACEi- and ARB-induced acute kidney injury (AKI) [11, 12]. However, no studies on the role of Sac/Val in dialysis patients with reduced EF have been conducted so far. In this prospective, single-blinded pioneer study, we evaluated the effects of Sac/Val on cardiac function in both HD and PD patients with HFrEF. Encouragingly, we demonstrated that Sac/Val increased EF in these patients without safety concerns. Concerning the safety issue, a recent meta-analysis [13] revealed that treatment with Sac/Val or omapatrilat (neprilysin+ACEi) showed a slightly lower risk of any renal event (odds ratio (OR) 0.82 (0.7-0.97)) compared with treatment with an ACEi or ARB alone, a decreased risk of severe acute renal events (OR 0.8 (0.69-0.93), and a decrease in estimated glomerular filtration rate decline (mean difference −0.58 mL/min). There was no difference in chronic renal events (OR 0.92 (0.8-1.05)) or hyperkalemia (OR 1.02 (0.84-1.23)), suggesting that neutral endopeptidase inhibitors (NEPi)+ ACEi/ARBs have a safe record at the renal level. In line with our findings, Kong et al. [14] demonstrated that administration of Sac/Val (50 mg twice a day) for one year improved the symptoms of HF and reduced NT-proBNP levels, accompanied by a decrease in blood pressure in a single HD patient with HFrEF. Although the authors attributed these cardiac beneficial effects to Sac/Val, it should be emphasized that the drug was given along with spironolactone at a dose of 20 mg three times a day and metoprolol at a dose of 23.75 mg once daily. In September 2022, Niu et al. [15] reported that treatment of 26 ESKD/HFrEF patients with Sac/Val (24 mg/26 mg twice daily and up-titration by doubling the dose every two to four weeks until a target dose of 97 mg/103 mg twice daily is reached) for one year improved EF from 31.3% to 45.1% (P <0.0001). These beneficial effects of Sac/Val on cardiac function were associated with a reduction in LVESV and LV internal diameter at the end-systole phase. These parameters were unchanged in 23 ESKD patients who received conventional treatment. The magnitude of improvement in EF is comparable to that observed in our study. Our study extended these findings by demonstrating that Sac/Val therapy reduced pulmonary hypertension. Moreover, we reported that Sac/Val improved quality of life at multi-aspect levels like physical limitations, symptoms, quality of life, and social limitations, where the summary score significantly improved (from 45.15 ± 5.04 to 71.84 ± 3.21, P = 0.00016). Similar results were reported by Kuwae [16], where the efficacy of Sac/Val (50/50 mg/day administered for six months) was tested in five HD patients with hypertension, including a patient with HFrEF and a patient with HFpEF. In agreement with our findings, the author demonstrated that Sac/Val significantly decreased the left atrial dimension and increased the LVEF from 58.2% ± 16.9% to 66.4% ± 15.0%, along with reduced NT-proBNP levels throughout six months.

The mechanisms underlying the cardioprotective effects of Sac/Val in HD patients with HFrEF are largely known. However, Sac/Val inhibits the degradation of BNP, a specific biomarker of HF that is produced and secreted by cardiac ventricles [17], which is known to play a beneficial role in both the cardiac and renal tissues in HF [18, 19], as evident by inducing vasodilation, diuresis, and sodium excretion, as well as anti-fibrotic and anti-inflammatory actions. In the current study, BNP levels were not significantly elevated following Sac/Val treatment, probably due to the cardioprotective action of the drug and the improvement of EF, which is characterized by a decline in BNP that may attenuate the increase in circulating levels of the hormone secondary to inhibition of its degradation. In addition, Sac/Val inhibits the deleterious effects of RAAS, which is involved in aggravating cardiac remodeling and renal damage in HF [20,21]. Specifically, angiotensin II is widely acknowledged to be associated with the progression of HF and the risk of death [22,23], so its elimination by Sac/Val may contribute to the beneficial effects of the drug on cardiac function in our patients. Finally, uremia is characterized by chronic inflammation and endothelial dysfunction. In this context, serum levels of cytokines, including interleukin (IL)-1β and tumor necrosis factor-alpha (TNF-α), and IL-6 are elevated in HD patients, suggesting an adverse role of these substances in the pathogenesis of cardiac failure in these patients [24]. Interestingly, Sac/Val treatment did not affect blood pressure in our patients after three months of treatment, where SBP and DBP were 139 ± 6 mmHg and 64 ± 3 mmHg, respectively, as compared with basal SBP and DBP of 143 ± 7 mmHg and 63 ± 3 mmHg. The lack of hypotensive effects of Sac/Val rules out the contribution of lowering blood pressure to the positive effects of the drug on EF. This notion is in agreement with experimental findings that the anti-cardiac hypertrophic effects of Sac/Val in an AngII-induced high blood pressure mouse model were independent of a blood pressure-lowering effect.

Despite these encouraging results, the current study suffers from a few limitations, which are as follows: a low sample size (n = 13), a short period of treatment, and a short follow-up period. We recommend increasing the number of patients and extending the follow-up period in a few studies.

## Conclusions

In summary, Sac/Val, a dual inhibitor/blocker of the neprilysin and angiotensin II receptors, exerts cardioprotective effects among patients with HFrEF. Unfortunately, the drug is not approved for subjects with advanced CKD or dialysis patients due to safety concerns. The current study examined the cardiac effects of Sac/Val in dialysis patients. Administration of Sac/Val to dialysis patients with HFrEF for three and six months improved EF by 50% independently of morphological changes in cardiac geometry and hemodynamic alterations. Likewise, the quality of life was dramatically improved following Sac/Val. No major adverse effects were reported in the present study, supporting the safety of Sac/Val in these high-risk patients.

Collectively, these findings support the use of Sac/Val as a cardioprotective agent in both hemodialysis and peritoneal dialysis patients. Larger prospective studies are warranted to investigate the impact of Sac/Val treatment on HF hospitalization and the survival rate of these dialysis patients in view of the high rate of cardiovascular death. 

## Appendices

Appendix A

Cardiomyopathy Questionnaire (Kansas City KCCQ-12)

The Cardiomyopathy Questionnaire (Kansas City KCCQ-12) was applied to assess changes in quality of life after Sac/Val as compared to pretreatment status (https://cvam.com/wp-content/uploads/2019/07/KCCQ-Questionnaire.pdf).
